# eIF4E and 4EBP1 are prognostic markers of head and neck squamous cell carcinoma recurrence after definitive surgery and adjuvant radiotherapy

**DOI:** 10.1371/journal.pone.0225537

**Published:** 2019-11-22

**Authors:** Chung-I. Huang, Chih-Chun Wang, Tzong-Shyuan Tai, Tzer-Zen Hwang, Chuan-Chien Yang, Chin-Mu Hsu, Yu-Chieh Su

**Affiliations:** 1 Department of Radiation Oncology, E-Da Cancer Hospital, Kaohsiung, Taiwan; 2 Department of Otolaryngology, E-Da Hospital, Kaohsiung, Taiwan; 3 Department of Medical Research, E-Da Hospital, Kaohsiung, Taiwan; 4 Division of Hematology-Oncology, Department of Internal Medicine, E-Da Hospital, Kaohsiung, Taiwan; 5 School of Medicine, I-Shou University, Kaohsiung, Taiwan; University of Wisconsin, UNITED STATES

## Abstract

There is high risk of metastasis and recurrence in head and neck squamous cell carcinoma (HNSCC) patients, especially for patient who received definitive surgery and adjuvant radiotherapy. Aberrant activation of PI3K/AKT/mTOR signaling occurs in approximately 80% of HNSCC, which has been indicated to serve as prognostic biomarkers for patients suffer from recurrence or metastasis. Therefore, in this study, we focus on the relationship between the expression level of signaling factors in PI3K/AKT/mTOR pathway and recurrence tumor from HNSCC patients. A tissue microarray (TMA) was constructed from 54 cases of HNSCC patients who received definitive surgery and adjuvant radiotherapy, are followed more than 5 years, and with no previous malignancy and synchronous tumor. Slides were scored and dichotomized by two pathologists and scores. Based on the TMA block with IHC staining, the results showed that PI3K/AKT/mTOR signaling was highly activated both in recurrence and non-recurrence patients. Particularly, in the recurrence population, the results showed the low expression phospho-eukaryotic initiation factor 4E (p-eIF4E) or high expression eIF4E, phospho-eIF4E binding protein 1 (p-4EBP1), phospho-ribosomal protein S6 kinase beta-1 (p-S6K1) and phospho-40S ribosomal protein S6 (p-S6R) exhibited worse overall survival. The expression level of eIF4E and p-4EBP1 were significantly associated with tumor recurrence and recurrence-free survival. Furthermore, high expression level of eIF4E and p-4EBP1 had worse recurrence-free survival. In conclusion, the expression of eIF4E and p-4EBP1 should be considered as predictive biomarkers for the HNSCC patients. This may contribute to potential predictive biomarkers for HNSCC patient who receive adjuvant radiotherapy.

## Introduction

Head and neck squamous cell carcinoma (HNSCC) includes cancers of the oral cavity, larynx, and oropharynx and is the sixth most common cancer worldwide [1]. Globally, HNSCC accounts for more than 809,000 incidence cases and 316,000 deaths, which comprises 3.6% of deaths from all cancers [2]. The rates of incidence cases and mortality due to HNSCC are different based on the geographical site and location, with a high incidence observed in South Asia and Southeast Asia [3]. The incidence rate of oral cancer in Taiwan is among the highest in the world, accounting for 8.0% of all new cancers and 6.3% of deaths from all cancers [4]. Several risk factors have been implicated in the etiology of HNSCC, such as alcohol, tobacco, and betel nut consumption [5].

Patients with HNSCC diagnosed in early stage (I and II) have a 60%–95% possibility of achieving successful treatment via primary tumor excision combined with extensive neck dissection, which results in a favorable outcome for long-term prognosis [6]. For patients with non-metastatic HNSCC, concurrent chemotherapy and the combination of chemotherapy and radiotherapy are the nonsurgical standard of care with good performance outcomes [7]. However, in two-thirds of diagnosed patients, the cancer is already in the advanced stage (III and IV) and is accompanied by tumor metastasis and recurrence, leading to poor prognosis and poor quality of life [8]. Recurrence rate in patients with HNSCC is approximately 50% within 2 years of the diagnosis of the primary tumor [9]. A previous study has reported that the most challenging issue in patients with HNSCC is the development of second primary tumors and primary tumor recurrence. The use of salvage surgery or reradiation is limited for patients with recurrence or metastasis [10]. Chemotherapy can be used for such patients, and it increases the response rates from 10% to 35% with a median survival of 6–12 months [11]. However, the use of concurrent radiotherapy or chemotherapy in the treatment of patients with HNSCC is accompanied by severe side effects, including mucositis, dysphagia, leukopenia, and thrombocytopenia, which leads to an increased risk of infection and bleeding [12].

The prediction of clinical and biological behavior of cancer is mainly dependent on the histological grading, and concerns have been raised regarding the use of TNM classification as the criteria for prognostic evaluation of HNSCC [13]. However, the TNM staging system remains the basis of classification for prognosis and determining treatment modalities while judging the condition of patients with clinical HNSCC, leading to patients receiving the same or similar treatment for different stages of HNSCC [14]. A previous study has demonstrated several clinicopathological characteristics implicated in prognosis, such as tumor thickness, surgical margin status, recurrence, and survival [15]. Therefore, it is imperative to identify reliable and potential markers that can help predict the prognosis of patients with HNSCC to distinguish the different risk categories in these patients.

Several studies have indicated that the PI3K/AKT/mTOR signaling pathway is implicated in multiple cancerous behaviors, including cellular differentiation, metastasis, angiogenesis, and apoptosis. Therefore, this pathway has emerged as an important therapeutic target for developing anticancer drugs against HNSCC [16]. Activation of the mammalian target of rapamycin (mTOR) is mediated by several signaling factors, including growth factors, nutrients, and cellular stress, which promote protein synthesis, ribosome biogenesis, cell proliferation and survival, metastasis, and angiogenesis through the activation of ribosomal protein S6 kinase 1 (S6K1) and eukaryotic initiation factor 4E (eIF4E)-binding protein 1 (4EBP1) [17–19]. Upon phosphorylation, 4EBP1 releases the bound translational helicase eIF4A, leading to the initiation of translation of a subset of mRNAs critical for cancer progression, including c-myc, cyclin D1, and matrix metalloproteinsase-9 [20–22]. A previous study demonstrated that p-S6 expression in the activation of the PI3K/AKT/mTOR pathway serves as a potential prognostic biomarker and a predictor of cancers with distant metastasis [23]. In addition, phosphorylation of mTOR has been indicated as a reliable biomarker closely associated with HNSCC incidence [24]. In particular, a recent study has described a genomic prediction model in clinical practice, which may help provide individualized management for treating patients with HNSCC [8].

The aim of this study was to evaluate the association between the expression of proteins in the PI3K/mTOR/eIF4 signaling pathway and the risk of recurrence in patients with HNSCC who have received radical surgery and adjuvant radiotherapy. Tissue microarray (TMA) with immunohistochemical (IHC) staining was used to analyze the tumor specimens of patients with HNSCC who had received definitive surgery and adjuvant radiotherapy. Based on semi-quantified scoring systems, we detected a higher expression level of eIF4E and 4EBP1 and a lower expression level of P-PI3K in patients with HNSCC who experienced recurrence than in those who did not. Moreover, patients with HNSCC with higher expression levels of eIF4E and 4EBP1 exhibited lower recurrence-free survival than those with lower expression levels.

## Materials and methods

### Patients and tissue samples

Tumor specimens were obtained from patients admitted for diagnosis and treatment to the E-DA Hospital between April 2011 and March 2016. Patients’ personal information, clinical data, and medical records were collected; the study was approved and reviewed by the Institutional Review Board of the E-DA Hospital. Written informed consent was obtained from all adult participants. Each sample was fixed with formalin, embedded in paraffin wax, and stored at RT. Patients fulfilling the following criteria were included in this study: those 1) who received adjuvant radiotherapy, 2) were followed up for >3 years, 3) who had no previous malignancy, 4) who had no synchronous tumor, and 5) who had not undergone any treatment. Finally, tumor specimens were obtained from patients with oral, oropharyngeal, or hypopharyngeal cancer. Table 1 summarizes the clinicopathological data of the 54 study patients retrieved from their medical records.

**Table 1:** Baseline characteristics of head and neck cancer patients, *n* = 54.

	Not recurred	Recurred	
Variables	*n* (%)	*n* (%)	p value
Age, years (Mean±SD)	52±8	52±10	0.882
Gender			
Male	28	26	
Female	0	0	
AJCC Stage			0.609
1	0 (0%)	1 (4%)	
2	1 (4%)	2 (8%)	
3	5 (18%)	6 (23%)	
4	21 (78%)	17 (65%)	
AJCC pT			0.421
T1	1 (4%)	1 (4%)	
T2	9 (32%)	14 (54%)	
T3	16 (21%)	3 (11%)	
T4	12 (43%)	8 (31%)	
AJCC pN			0.722
N0	8 (29%)	6 (23%)	
N1	8 (29%)	6 (23%)	
N2	12 (42%)	14 (54%)	
Differentiation grade			0.036
well	2 (7%)	9 (34%)	
moderate	23 (82%)	16 (62%)	
poor	3 (11%)	1 (4%)	
PNI			0.051
positive	2 (7%)	7 (27%)	
negative	26 (93%)	19 (73%)	
LVI			0.423
positive	5 (18%)	7 (27%)	
negative	23 (82%)	19 (73%)	
Surgical margin			0.957
positive	1 (4%)	1 (4%)	
negative	27 (96%)	25 (96%)	
ECE			
positive	6 (21%)	11 (42%)	0.099
negative	22 (79%)	15 (58%)	

### TMA construction

After evaluation of hematoxylin–eosin tissue sections of each case, representative neoplastic areas were marked, and the corresponding paraffin block was retrieved. A tissue core 1.5 mm in diameter was obtained from each selected block using a manual arrayer (Model I; Beecher Instruments, San Prairie, WI, USA). Each TMA block contained 30 tissue cores, and multiple neoplastic and nonneoplastic tissue cores were obtained as assay controls.

### Immunohistochemistry

IHC staining was performed according to standard protocols. Briefly, 4-μm thick sections were cut from each TMA block and dewaxed in xylene, rehydrated through graded concentrations of ethanol, and placed in an antigen retrieval solution (citrate buffer, pH 6.0) for 15 min at 95°C. After cooling for 30 min, the tissue sections were incubated in 3% hydrogen peroxide for 10 min to block endogenous peroxidase activity. The slides were then washed thoroughly with phosphate-buffered saline and incubated (120 min, room temperature) with specific antibodies, including anti-PI3K p85/p55 (1:100, Arigo Biolaboratories Corp, Taiwan), anti-p-AKT (S473) (1:100, Abcam, MA, USA), anti-p-mTOR (S2448) (1:100, Abcam, MA, USA), anti-p-4EBP1 (Thr37/46) (1:400, Abcam, MA, USA), anti-p-S6K1 (Ser411) (1:100, Abcam, MA, USA), anti-p -S6 Ribosomal (Ser235/236) (1:400, Cell Signaling Technology, MA, USA), anti-p-eIF4E (S209) (1:500, Abcam, MA, USA), and anti-eIF4E (1:100, Cell Signaling Technology, MA, USA). Labeling was detected using Liquid DAB Substrate-Chromogen System (Dako North America Inc, Carpinteria, CA, USA) according to the manufacturer’s protocol. Sections were counterstained with hematoxylin, dehydrated, cleared, and mounted. Negative control was prepared by replacing the primary antibody with phosphate-buffered saline.

### Semi-quantified scoring systems

Using a subset of specimens, two pathologists separately scored the samples in a blinded manner, and the interrater reliability was assessed. The staining results were scored according to the semi-quantified scoring system described previously [25]. Briefly, as shown in Table 2, a score of “1” was assigned for strong nuclear staining in 1%–10% of the epithelium, a score of “2” was assigned for 11%–50%, a score of “3” was assigned for 51%–80%, and a score of “4” was assigned for 81%–100%. Intensity was scored according to the staining intensity as follows: score 0, no staining; score 1, weal staining; score 2, moderate staining; and score 3, strong staining. The sum score of intensity and extent of staining was multiplied for two parameters, resulting in an individual immunoreactivity score ranging between 0 and 12 for every case. Examples of staining intensity are shown in S1 Fig. In case of discrepancies, both pathologists used the same microscope to establish a final score by reassessment.

**Table 2:** Semi-quantified scoring system for IHC staining.

extent		intensity	
percentage of stained	score	intensity of staining	score
0% no cells stained	0	negative	0
1–10% of cells stained	1	weak	1
11%-50% of cells stained	2	moderate	2
51%-80% of cells stained	3	strong	3
81%-100% of cells stained	4		

### Statistical analysis

The sum score of intensity and extent of staining was grouped into low or high category (Table 3). Data of selected patient and tumor characteristics were obtained from medical records and presented in a frequency table using descriptive statistics. All data were presented as mean ± standard deviation. Significance levels were calculated using Student’s *t*-test, and *P* values < 0.05 were considered to be significant. Analysis was performed using the SPSS software for Windows (version 15.0; IBM Corporation, NY, USA).

**Table 3:** The sum score of intensity and extent of staining.

	Low	High
P-PI3K	0–9	10–12
P-AKT	0–5	6–12
P-mTOR	0–7	8–12
P-4EBP1	0–3	4–12
P-eIF4E	0–8	9–12
eIF4E	0–5	6–12
P-S6K	0–5	6–12
P-S6R	0–8	9–12

## Results

### Patient population and characteristics

Patients with HNSCC who received definitive surgery and adjuvant radiotherapy were included in the present study. Those who had a previous malignancy or a synchronous tumor at the time of diagnosis and who were followed up for <3 years were excluded. After applying the selection criteria and excluding cases with inadequate tissue for staining, 54 patients were included in this study; they were divided into two groups: 28 patients with non-recurrence and 26 with recurrence. As shown in Table 1, no significant differences were found between patients with or without recurrence in terms of age (*P* = 0.882), the American Joint Committee on Cancer (AJCC) stage (*P* = 0.609), AJCC pT (*P* = 0.421), and AJCC pN (*P* = 0.722). Several studies have demonstrated that differentiation grade, perineural invasion (PNI), and lymphovascular invasion (LVI) are implicated in poor survival and/or recurrence in patients with cancers [26]. Based on the histological assay, 9 (34%) tumors were found to be well-differentiated, 16 (62%) moderately differentiated, and 1 (4%) poorly differentiated in the group of patients with recurrence, which was significantly different from the group of patients without recurrence (*P* = 0.036). There were seven (27%) PNI-positive patients in the recurrence group, which was significantly higher than that in the non-recurrence group, with only 2 (7%) patients being PNI-positive. However, LVI showed no significant difference between the recurrence and non-recurrence groups. Moreover, the proportion of surgical margin and extracapsular extension showed no significant difference between the two groups.

### Clinicopathological parameters, recurrence, and survival

**Fig 1:**
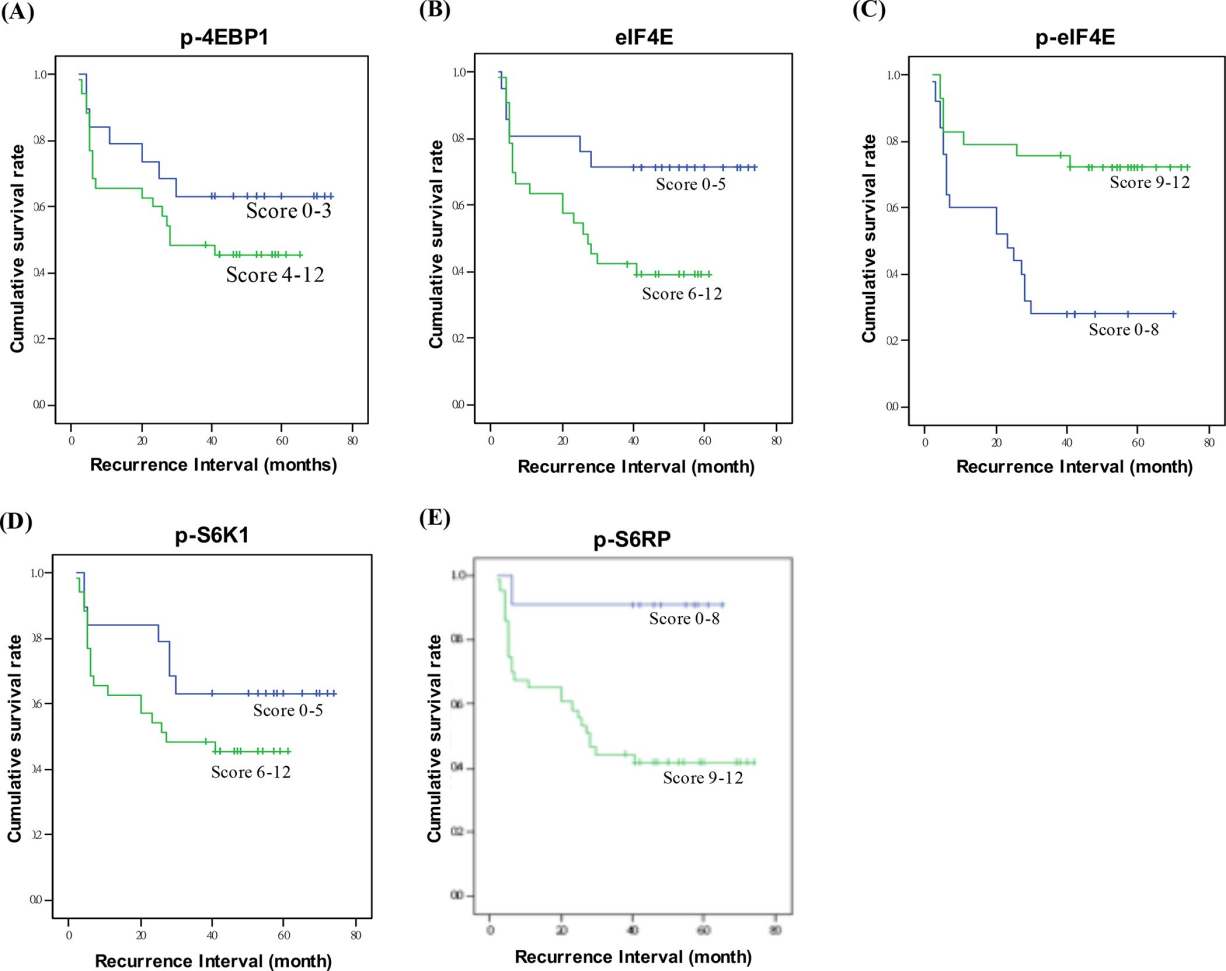
The expression of p-4EBP1, eIF4E, p-eIF4E, p-S6K, and p-S6RP were associated with cumulative survival rate in HNSCC patients with recurrence. Patients were grouped followed the sum score of intensity and extent of IHC staining. Kaplan–Meier plot was used to analyze the cumulative proportion of overall survival rate.

In the subgroup analysis, expression levels of eIF4E, p-PI3K, p-AKT, p-mTOR, p-4EBP1, p-eIF4E, p-S6K1, and p-S6R in the tumor specimens of patients with or without recurrence were analyzed using IHC staining and quantified by semi-quantified scoring systems. Results of the scoring systems revealed that all markers exhibited high expression in both the groups (Table 4), indicating that the PI3K/AKT/mTOR signaling pathway was highly activated in patients with HNSCC. On comparing the specimen scores of the non-recurrence group with the recurrence group, it was found that expression levels of eIF4E, p-4EBP, p-S6K1, and p-S6R were higher in the latter than in the former, whereas the scores for p-PI3K and p-eIF4E were lower in the latter (Table 4). To evaluate the association between the cumulative survival rate and expression levels of p-4EBP, eIF4E, p-eIF4E, p-S6K1, and p-S6R in patients with recurrence, we used the Kaplan–Meier plot. We found that patients with higher expression levels of p-4EBP1, eIF4E, p-S6K1, and p-S6RP (Fig 1A, 1B, 1D and 1E) or a lower expression level of p-eIF4E (Fig 1C) exhibited worse cumulative survival rate.

**Table 4:** IHC score means.

IHC	Not recurred	Recurred	p value
P-PI3K	11.4	9.9	<0.001
P-AKT	6.4	6.0	0.611
P-mTOR	7.9	8.6	0.138
P-4EBP1	3.7	5.7	0.281
eIF4E	5.7	7.7	0.701
P-eIF4E	10.4	8.4	0.794
P-S6K1	6.3	6.9	0.568
P-S6R	8.5	10.7	0.001

**Fig 2:**
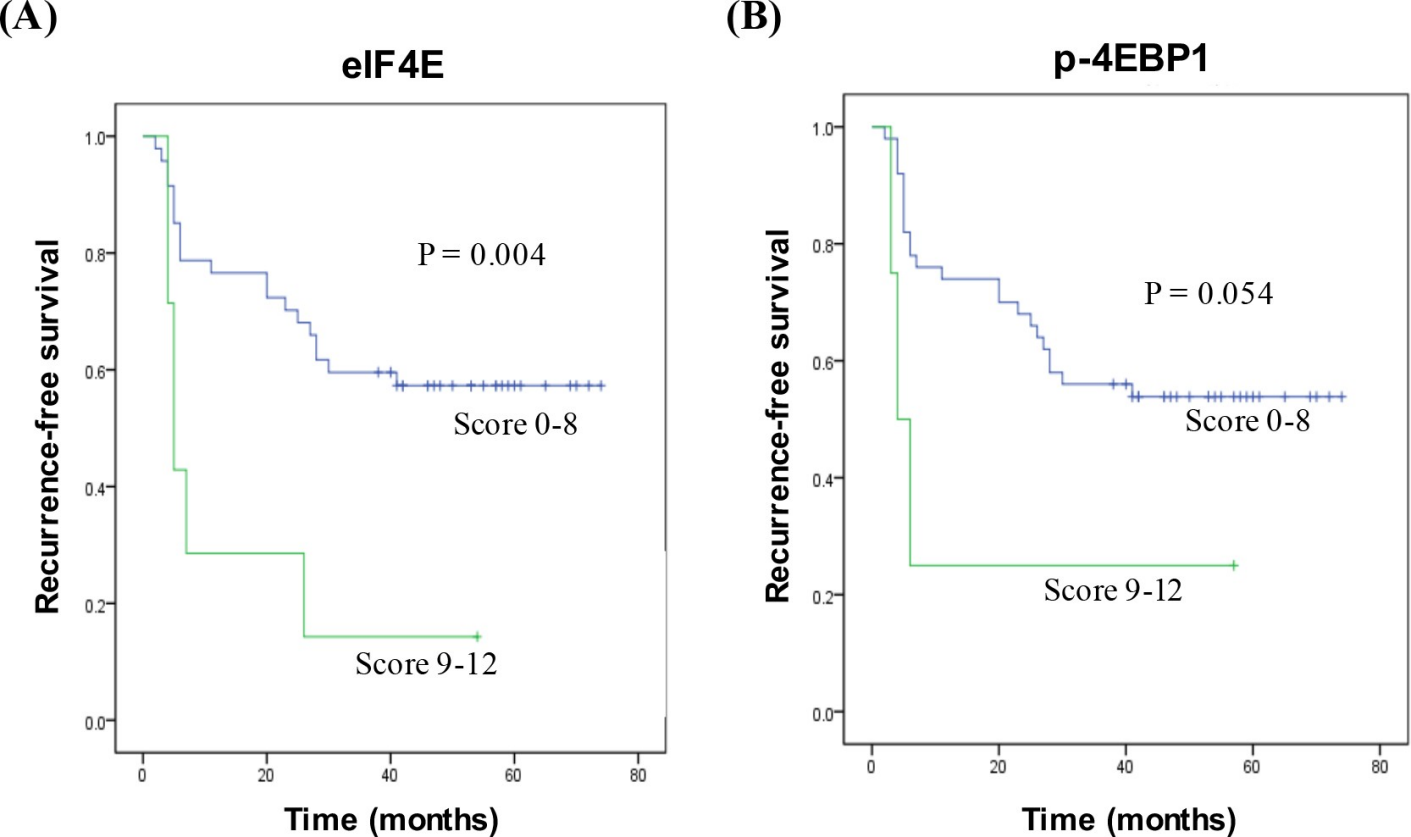
The expression of eIF4E and p-4EBP1 correlated with recurrence-free survival in HNSCC patients with recurrence. Patients were grouped followed the sum score of intensity and extent of IHC staining. Kaplan–Meier plot was used to analyze the cumulative proportion of recurrence-free survival.

**Fig 3:**
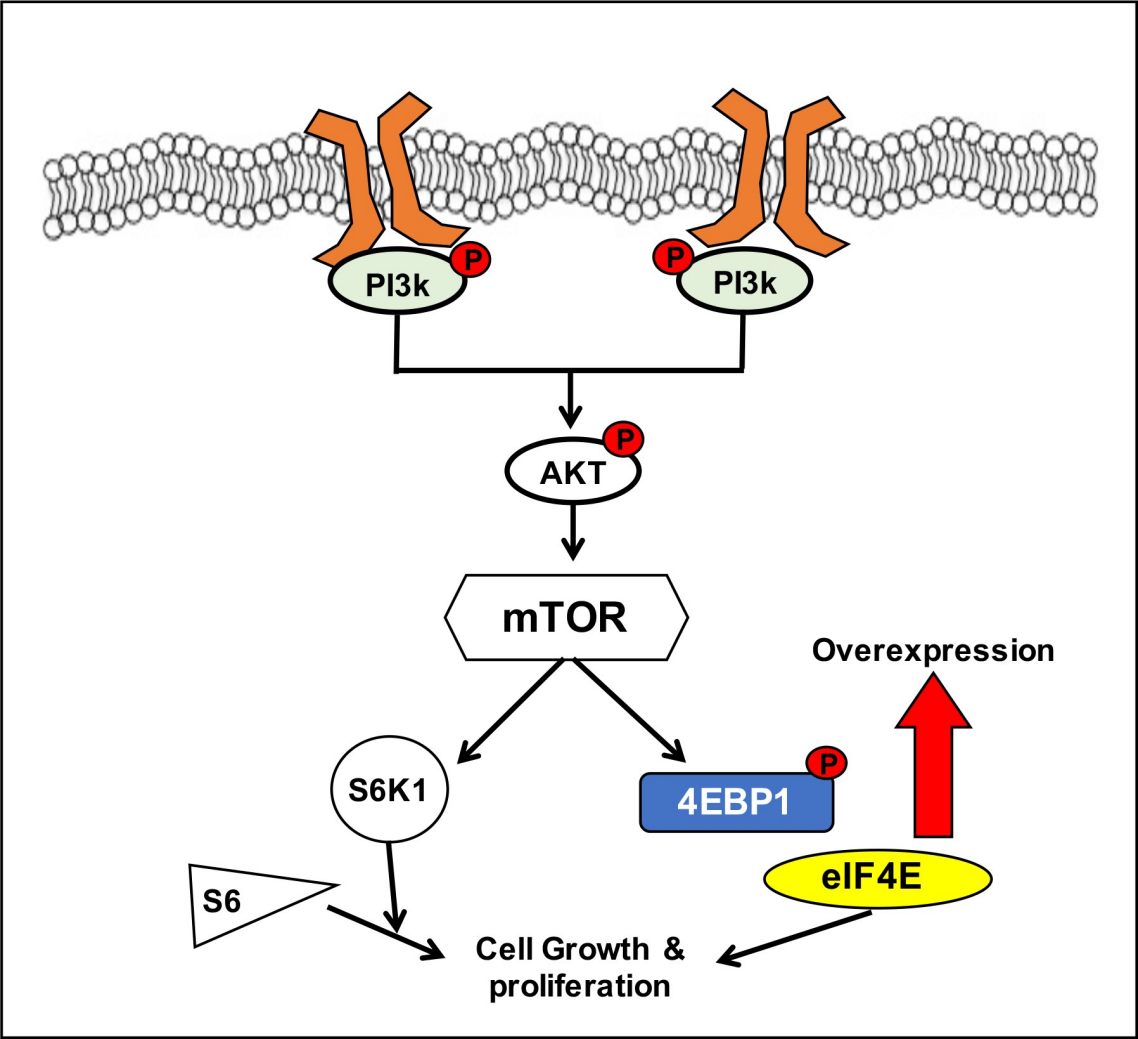
The schematic of overexpressed 4EBP1 and eIF4E as prognostic markers in the PI3K/AKT/mTOR pathway for patients with HNSCC.

We used multivariate analysis to investigate the association between recurrence-free survival and expression levels of p-eIF4E, eIF4E, and p-4EBP-1 in the patients with tumor recurrence. Results indicated that higher expression levels of eIF4E and p-4EBP-1 were associated with the risk for tumor recurrence, with hazard ratios of 4.1 (*P* = 0.011) and 16.6 (*P* = 0.001), respectively (Table 5). We further found that a higher expression level of p-eIF4E showed a weak association with tumor recurrence, with a hazard ratio of 0.173 (*P* ≤ 0.001). The Kaplan–Meier plot analysis revealed that patients with higher expression levels of eIF4E and p-4EBP-1 had worse recurrence-free survival than those with lower expression levels (Fig 2). The results indicated that high expression levels of eIF4E and p-4EBP1 had the potential to become predictive biomarkers for patients with HNSCC who receive adjuvant radiotherapy (Fig 3).

**Table 5:** Multivariate analysis for recurrence free survival.

Markers	βcoefficient	Hazard ratio	95% confidence interval	p value
P-eIF4E				
high (9–12)	-1.755	0.173	0.067–0.447	<0.001
low (0–8)		1		
eIF4E				
high (9–12)	1.415	4.115	1.389–12.185	0.011
low (0–8)		1		
P-4EBP1				
high	2.806	16.55	2.984–91.781	0.001
low		1		

## Discussion

In this study, we determined expression levels of signaling factors in the PI3K/AKT/mTOR pathway in patients with HNSCC with or without recurrence who had previously received definitive surgery and adjuvant radiotherapy. We found high expression levels of p-PI3K, p-AKT, p-mTOR, p-4EBP-1, p-eIF4E, and eIF4E in the specimens of both the recurrence and non-recurrence groups (Table 1). Studies have revealed a similar phenomenon in patients with HNSCC, indicating that overexpressed proteins in the PI3K/AKT/mTOR signaling pathway may be used as good prognostic biomarkers, including p-mTOR, p-PI3K, and p-S6 [27]. However, only a few studies have investigated the prognostic markers for predicting the risk of recurrence in patients with HNSCC who have undergone definitive surgery and adjuvant radiotherapy. In the present study, we found that patients with tumor recurrence had higher expression levels of p-4EBP-1, eIF4E, and p-S6R and lower expression levels of p-PI3K and p-eIF4E than those without tumor recurrence, indicating that these signaling factors of the PI3K/AKT/mTOR pathway may worth investigating as the potential biomarkers for tumor recurrence prognosis.

Previous studies have demonstrated the overexpression of PI3K/AKT in various cancers, which has been indicated as a potential prognostic biomarker [28]. An earlier study also indicated that the expression of p-eIF4E, but not eIF4E, served as a predictor for survival in male breast cancer [29]. In our study, we observed lower expression levels of p-PI3K and p-eIF4E in patients with tumor recurrence than in those without recurrence. These results indicate that p-PI3K and p-eIF4E may also be used as prognostic markers for predicting recurrence and need to be investigated in further studies. Moreover, the underlying mechanism of lower expression levels of p-PI3K and p-eIF4E in patients with tumor recurrence must be investigated.

Based on the results of Kaplan–Meier plot analysis, we found that the lower expression level of p-eIF4E or higher expression levels of eIF4E, p-4EBP, p-S6K1, and p-S6R were associated with worse cumulative survival in patients with tumor recurrence (Fig 1), which indicated that these protein levels correlated with the survival of these patients. A previous study has demonstrated elevated levels of lactate in HNSCC tumors, which correlated with the overall survival of the patients [30]. Moreover, the authors of that study found that high expression levels of lactate in the primary tumor were associated with tumor recurrence in patients with HNSCC who had received radiotherapy [30]. Another study demonstrated that genetic variations at important loci within the PI3K/PTEN/AKT/mTOR pathway could be used to identify patients with high risk of tumor recurrence or second primary tumor occurrence [28]. Furthermore, another study provided a genomic prediction model for tumor recurrence and metastasis development in patients with HNSCC by comparing the genomic hybridization data comprising the signaling pathway of cell proliferation and invasion [8]. With all these potential prognostic markers for predicting the incidence of recurrence in patients with HNSCC, the association of these prognostic markers is worth evaluating for providing a more personalized clinical management to patients with HNSCC.

The PI3K/AKT/mTOR signaling pathway has been indicated to be implicated in the development, progression, and metastasis of numerous cancers [31]. In this pathway, the translation initiators that influence the downstream protein synthesis include eIF4E, 4EBP1, and S6K, and the rate of protein synthesis is dependent on the status of phosphorylation [32]. In the present study, we found that expression levels of eIF4E, p-4EBP1, p-eIF4E, p-S6K1, and p-S6R were elevated in both the patients with recurrence and non-recurrence, which may indicate that cell growth and proliferation rates are higher in patients with tumor recurrence than in those without recurrence. Correspondingly, elevated expressions of eIF4E, p-4EBP1, and S6K1 correlated with breast cancer proliferation and survival [33]. Moreover, a strong relationship between MNK-mediated phosphorylation of eIF4E has been demonstrated in prostate and breast cancers [34]. As the phosphorylation of eIF4E, 4EBP1, and S6K is known to affect the rate of translation initiation, it is possible that these proteins play a critical role in the progression of HNSCC. In further studies, an *in vitro* or *in vivo* analysis using specific inhibitors should be performed to evaluate the importance of these proteins in the progression of HNSCC along with the profile of IHC staining observed in the present study.

## Supporting information

S1 Fig The scoring system example for manual semi-quantitative evaluation. (TIF)
