# Supplementary figures and images for: eIF4E and 4EBP1 are prognostic markers of head and neck squamous cell carcinoma recurrence after definitive surgery and adjuvant radiotherapy
Source: PLoS One. 2019 Nov 22;14(11):e0225537. doi: 10.1371/journal.pone.0225537 (PMC6874317; doi:10.1371/journal.pone.0225537)

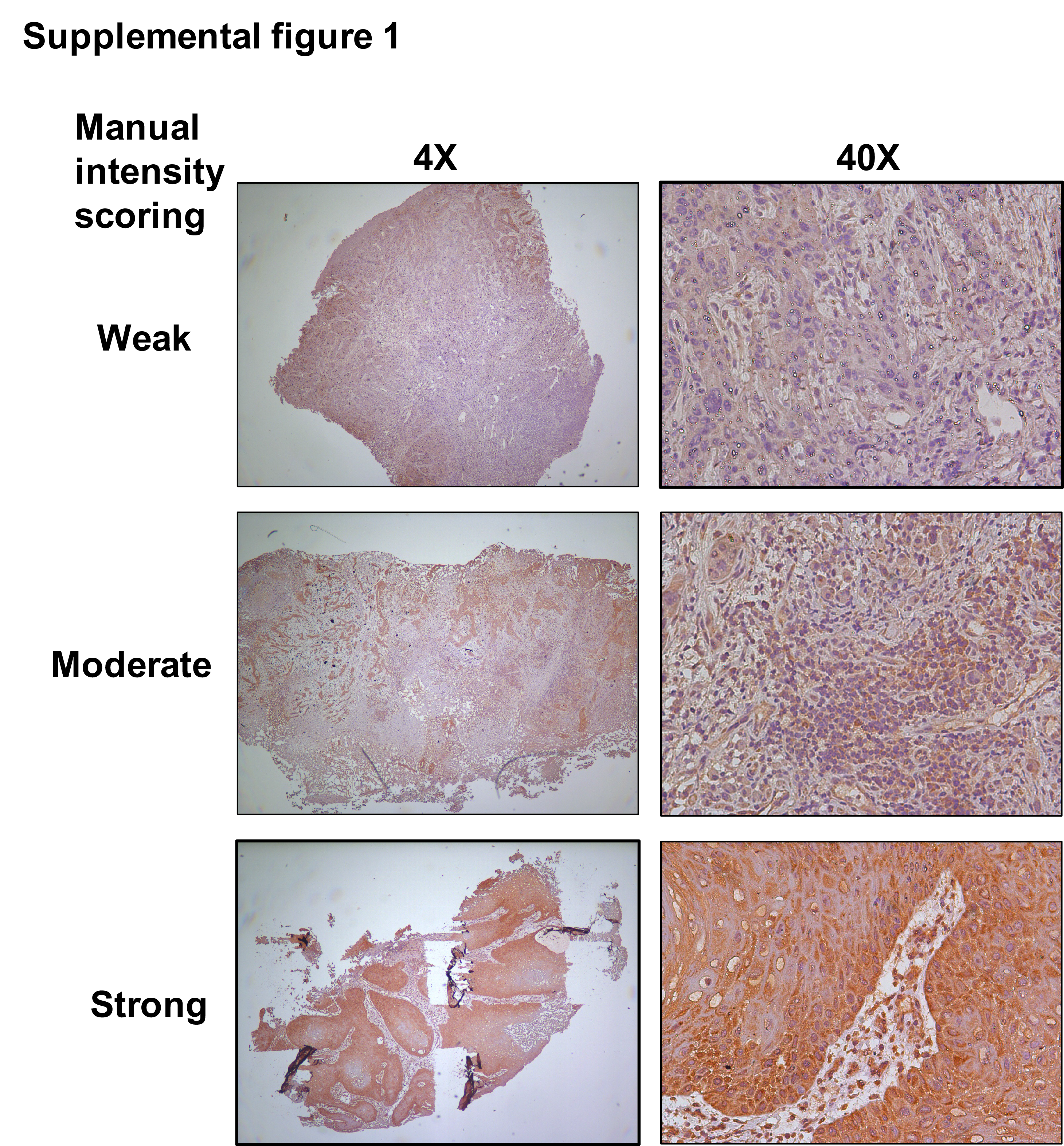

Supplement: S1 Fig — (TIF) [file pone.0225537.s001.tif]
